# What is the value of multidisciplinary care for chronic kidney disease?

**DOI:** 10.1371/journal.pmed.1002533

**Published:** 2018-03-27

**Authors:** Richard J. Fluck, Maarten W. Taal

**Affiliations:** 1 Department of Renal Medicine, Royal Derby Hospital, Derby, United Kingdom; 2 Centre for Kidney Research and Innovation, Division of Medical Sciences and Graduate Entry Medicine, University of Nottingham, Derby, United Kingdom

## Abstract

In a Persepctive, Richard Fluck and Maarten Taal discuss the potential value of implementing multidisciplinary care programs for chronic kidney disease.

Chronic kidney disease (CKD) is associated with multiple biochemical and physiological abnormalities as well as important adverse outcomes that include increased risk of cardiovascular events (CVEs) [1], acute kidney injury (AKI) [2], and progression to end-stage kidney disease (ESKD). These factors in turn impact negatively on quality of life and increase the burden on healthcare resources. In this context, multidisciplinary care (MDC), advocating a holistic approach to treatment, has the potential to improve care and reduce the risk of adverse outcomes. Importantly, timely referral to multidisciplinary nephrology services allows effective preparation for ESKD, including evaluation for kidney transplantation, dialysis modality selection, dialysis access planning, and financial, social, and psychological support. However, the provision of MDC requires additional funding and personnel, and it is therefore vital that advocates of MDC provide evidence of benefit and value to justify the additional cost. This is particularly important because CKD consumes a disproportionate share of healthcare funding globally due to the need for long-term care, a high rate of medical complications, and the high cost of renal replacement therapy (RRT) [3].

The publication this week in *PLOS Medicine* of a comprehensive model to assess the cost-effectiveness of MDC for patients with CKD, by Eugene Lin and colleagues [4], is therefore a welcome development. The authors used data from published studies and Medicare claims in the United States to develop a novel Markov model of CKD progression and used this to assess the impact of a theoretical programme of MDC in mild to moderate CKD (estimated glomerular filtration rate [eGFR] 20–59 ml/min/1.73m^2^), with the intention of assessing the effectiveness of MDC in slowing progression of CKD and reducing mortality. Predicted benefit was measured as quality-adjusted life years (QALYs) gained. Overall, MDC was estimated to add 0.23 QALYs (95% CI 0.08–0.42) over usual care at a cost of US$51,285 per QALY gained, within generally accepted cost-effectiveness thresholds for high-income countries. Robust sensitivity testing showed that MDC remained cost-effective at a threshold of US$150,000 per QALY gained, even when effectiveness was reduced to 25% or cost was increased 5-fold. This analysis will be very useful to providers and those responsible for commissioning and funding services seeking to implement MDC for CKD, but the paper also highlights important areas of uncertainty that require further exploration.

First, what constitutes optimal MDC for CKD? In their analysis, the authors considered MDC aimed at reducing CKD progression and made reasonable assumptions to include components delivered by a nephrologist or advanced practitioner, a CKD educator, a dietician, and a social worker. It is clear, however, that additional components could have been added, including input from a pharmacist, a physiotherapist, an occupational therapist, or a psychologist. The optimal composition of MDC will likely vary with the stage and severity of CKD as well as the target for improvement, but this remains to be determined. This consideration is critical because each component adds cost and may have differing impact on the benefits. In the National Health Service (NHS) in England, reimbursement for nephrology outpatient attendance has an enhanced tariff payment for MDC. At present, this tariff applies only to people with advanced CKD attending clinics intended to treat complications and prepare them for RRT. The tariff does not specify the individual components of the service and allows providers to determine their best configuration, but as yet, there has been no evaluation against defined metrics. Further research is therefore required to evaluate the optimal and most cost-effective composition of MDC for different stages of CKD.

Second, this paper illustrates that the cost-effectiveness of MDC varies with the risk profile of patients. MDC was most cost-effective in patients with the highest level of albuminuria because of their higher risk of CKD progression and, therefore, greater benefit from intervention. Nevertheless, younger patients and, perhaps counterintuitively, those with less albuminuria achieved the greatest increase in QALYs, likely reflecting their longer life expectancy. MDC remained cost-effective in these latter groups, though relatively more expensive because care would have to continue longer to achieve benefit. Patients without albuminuria are at low risk of progression to ESKD, and it is therefore likely that most of the benefit in this group resulted from reduction in risk of cardiovascular death, a finding confirmed in a sensitivity analysis that assumed no effect in reducing ESKD. These people would therefore benefit from MDC focused on cardiovascular risk reduction and could be spared the components directed at slowing CKD progression.

On the other hand, patients with advanced CKD would likely benefit from comprehensive MDC to address complications such as anaemia and facilitate timely preparation for RRT. Despite the robust findings of cost-effectiveness, it is likely that patients at low risk for CVEs and ESKD will not benefit from MDC at all. These considerations illustrate the need to stratify patients according to risk and develop packages of MDC that are best suited to their risk profile and CKD stage. This would result in more cost-effective use of MDC and would reduce the total cost burden. Components of MDC, particularly for lower-risk patients, would likely be best delivered in primary care as part of broader chronic disease management programmes, further lowering the cost.

Finally, the paper by Lin and colleagues focused on the benefit of MDC in reducing progression to ESKD and improving survival but did not consider potential improvement in several other important outcomes, including CVEs, hospital admissions, and episodes of AKI. For many patients with CKD, the risk of a CVE is substantially greater than the risk of progression to ESKD, so this is an important consideration [1]. Additionally, the analysis did not consider the impact on aspects likely to be of more immediate relevance to patients, including quality of life, patient-reported outcome measures, and a patient’s level of engagement in care [5]. This is largely because few studies evaluating these outcomes have been published and because cost-effectiveness by these criteria is more difficult to assess, but ironically, it is in these areas that MDC may afford the greatest benefit. Thus, Lin and colleagues’ study likely underestimated the full value of MDC and highlights the need for more research to investigate benefit with respect to all outcomes.

As acknowledged by the authors, this paper presents a theoretical model based on limited currently available data, but the study provides robust evidence that MDC for CKD is cost-effective, even with pessimistic assumptions. Much remains to be done to identify the optimal package of care for different patient subgroups, but we agree with the authors that the data provide sufficient evidence to support initiation of pilot MDC programmes as well as further research to identify optimal models for implementation. Critically, future studies should collect metrics that comprehensively assess the benefit of MDC and that are aligned with international initiatives such as Standardised Outcomes in Nephrology (SONG) [6] (http://songinitiative.org) and include patient-determined measures.

## Abbreviations

AKI: acute kidney injury
CKD: chronic kidney disease
CVE: cardiovascular event
eGFR: estimated glomerular filtration rate
ESKD: end-stage kidney disease
MDC: multidisciplinary care
NHS: National Health Service
QALY: quality-adjusted life year
RRT: renal replacement therapy
SONG: Standardised Outcomes in Nephrology
